# Supplementary material for: Estimated impact of the UK soft drinks industry levy on childhood hospital admissions for carious tooth extractions: interrupted time series analysis
Source: BMJ Nutr Prev Health. 2023 Nov 14;6(2):243–52. doi: 10.1136/bmjnph-2023-000714 (PMC10800259; doi:10.1136/bmjnph-2023-000714)
Supplement: Supplementary data [file bmjnph-2023-000714supp001.pdf]

Table S1: Coefficients of interaction between study phases, IMD and age-groups.

	Estimate[1]	Standard error	P-value
**IMD fifths**			
Phase: Pre-announcement			
IMD1 (least deprived)	ref	ref	ref
IMD2	0.01	0.03	0.67
IMD3	-0.01	0.03	0.67
IMD4	0.02	0.03	0.44
IMD5 (most deprived)	-0.06	0.08	0.03*
Phase: Post-announcement - pre-implementation			
IMD1 (least deprived)	ref	ref	ref
IMD2	-0.03	0.08	0.71
IMD3	-0.04	0.08	0.60
IMD4	-0.19	0.08	0.01*
IMD5 (most deprived)	0.04	0.08	0.61
Post-implementation phase			
IMD1 (least deprived)	ref	ref	ref
IMD2	0.01	0.12	0.92
IMD3	0.06	0.12	0.64
IMD4	0.13	0.12	0.29
IMD5 (most deprived)	-0.18	0.12	0.14
**Age groups**			
Phase: Pre-announcement			
0-4 years	ref	ref	ref
5-9 years	-0.10	0.03	0.0007*
10-14 years	-0.05	0.03	0.07
15-18 years	-0.04	0.03	0.21
Phase: Post-announcement - pre-implementation			
0-4 years	ref	ref	ref
5-9 years	0.17	0.08	0.04*
10-14 years	0.14	0.08	0.07
15-18 years	0.14	0.08	0.09
Phase: Post-implementation			
0-4 years	ref	ref	ref
5-9 years	-0.2	0.13	0.13
10-14 years	<0.01	0.13	1.00
15-18 years	-0.01	0.13	0.93

[1]Change in incidence rate (n/100,000 population/month)

Table S2: Changes[1] in incidence/100,000 population/month of hospital admissions for carious tooth extractions (95% confidence intervals), overall and by Index of multiple deprivation (IMD) fifth and age group at 22 months post-implementation of the UK SDIL.

	Absolute change	Relative change (%)
Total population	**-3·7(-2.2, -5·2)**	**-12.1(-17·0, -7·2)**
Deprivation fifths		
IMD1 (most deprived)	**-3·1(-0·4, -5·7)**	**-5·4(-10·0, -0·75)**
IMD2	**-6·6(-4·4, -8·8)**	**-16·8(-22·4, -11·3)**
IMD3	-1·6(0·49, -3·62)	-6·8(-15·6, 2·1)
IMD4	**-2·0(-1·06, -2·9)**	**-11·7(-17·2, -6·2)**
IMD5 (least deprived)	**-0·9(-0·2, -1·5)**	**-7·2(-12·5, -1·9)**
Age Group (years):		
0-4	**-6·5(-4·9, -8·1)**	**-28·6(-35·6, -21·5)**
5-9	**-3·3(-0·3, -6·4)**	**-5·5(-10·5, - 0·5)**
10-14	0·2(1·3, -0·8)	1·21(-4·4, 6·8)
15-18	-0·41(0·39, -1·2)	-4·95(-14·5, 4·62)

[1]Absolute and relative changes compared to the counterfactual scenario, which is based on a scenario of no SDIL announcement and no implementation

Table S3: Changes[1] in incidence /100,000 population/month of hospital admissions for carious tooth extractions (95% confidence intervals), overall and by Index of multiple deprivation (IMD) fifth and age group at 22 months post SDIL implementation (secondary analysis).

	Absolute change	Relative change (%)
Total population	1·32(2·8, -0·19)	5·11(-0·7, 11·0)
Deprivation fifths		
IMD1 (most deprived)	**-3·3(-0·7, -6·0)**	**-5.8(-10·4, -1·2)**
IMD2	**3·95(6·1, 1·8)**	**13.7(6·1, 21·3)**
IMD3	**2·1(4·18, 0·07)**	**10.9(0·36, 21·4)**
IMD4	0·3(1·2, -0·7)	1.9(-4·4, 8·2)
IMD5 (least deprived)	-0·1(0·6, -0·7)	-0.8(-6·4, 4·9)
Age group (years)		
0-4	**2·5(4·1, 0·9)**	**18·6 (6·9, 30·2)**
5-9	-0·7(2·4, -3·8)	-1·1(-6·4, 4·1)
10-14	**2·5(3·6, 1·5)**	**15·4(9·0, 21·8)**
15-18	**1·9(2·7, 1·1)**	**30·6(17·4, 43·7)**

[1]Absolute and relative changes compared to the counterfactual scenario, which is based on a scenario of an SDIL announcement but no implementation

Changes to Protocol

Several substantive changes were made to the published protocol. Study outcomes were initially planned to be by age and deprivation and then further by gender. However, cases per 100,000 population of hospital admissions for dental extraction for dental caries each month, were deemed too small to further stratify by gender. For the same reason, our original plans to examine hospital admission by IMD tenths was revised to IMD fifths. It was planned that acute cases of asthma or appendectomy could be used as a control group however relatively unstable incidence rates by deprivation and during the study period made these conditions unsuitable as controls. The study period was ended two months earlier than planned because of the national lockdown due to the COVID-19 pandemic which began in March 2020.
